# Alkhurma Hemorrhagic Fever in Travelers Returning from Egypt, 2010

**DOI:** 10.3201/eid1708.101858

**Published:** 2011-08

**Authors:** Rémi N. Charrel, Ernest A. Gould

**Affiliations:** Author affiliations: Université de la Méditerranée, Marseille, France (R.N. Charrel, E.A. Gould);; National Environment Research Council, Center for Ecology & Hydrology, Oxford, UK (E.A. Gould)

**Keywords:** viruses, hemorrhagic fever, Alkhurma hemorrhagic fever, travelers, letter

**To the Editor:** The report of 2 visitors from Italy being infected by Alkhurma hemorrhagic fever virus (AHFV) in southeastern Egypt near the border with Sudan (*1*) provides useful data to help clarify the evolutionary origin of these tick-borne flaviviruses. AHFV was first isolated in Saudi Arabia and is associated with camel ticks (*2*). It is a genetically close relative of Kyasanur Forest disease virus, which was first isolated in India in 1957. Following the original isolation of Kyasanur Forest disease virus, there was no clear explanation for its apparent isolation in the Indian forests. Indeed, its subsequent discovery in southern China (*3*) suggested that migratory birds might carry the infected ticks to or from that region.

The most likely explanation for these outbreaks of hemorrhagic disease now begins to fit a pattern that can be interpreted in terms of the diseases’ evolutionary origin in Africa. Thousands of animals are annually transported from Africa and other countries to Mecca, Saudi Arabia, to meet the human demand for food and transport during the Hajj. Many of these animals, including camels, are infested with ticks that may carry AHFV and thus provide the source of this human infectious agent. Phylogenetic evidence had previously suggested that the tick-borne encephalitic flavivirus serocomplex originated in Africa and gradually evolved and dispersed across the Northern Hemisphere of the Old World (*4**,**5*). This concept is totally consistent with the discoveries of AHFV in Saudi Arabia and now in southeastern Egypt. Thus, Africa is a likely source of infected ticks that are regularly moved between Africa and Saudi Arabia. This concept of an African evolutionary origin for these viruses could readily be tested by serologic investigation of humans and animals and also by analysis of ticks from this region of Africa.
