# Supplementary material for: Alkhurma Hemorrhagic Fever in Travelers Returning from Egypt, 2010
Source: Emerg Infect Dis. 2011 Aug;17(8):1573–4. doi: 10.3201/eid1708.101858 (PMC3381587; doi:10.3201/eid1708.101858)
Supplement: Related texts

# Reply: Alkhurma Hemorrhagic Fever in Travelers Returning from Egypt, 2010

**Authors:** Fabrizio Carletti, Concetta Castilletti, Antonio Di Caro, Maria R. Capobianchi, Carla Nisii, Fredy Suter, Marco Rizzi, Alessandra Tebaldi, Antonion Goglio, Cristiana Passerini Tosi, Giuseppe Ippolito

**Affiliations:** Author affiliation: Lazzaro Spallanzani National Institute for Infectious Diseases, Rome, Italy

**In Response:** The concept suggested by Charrel and Gould (*1*) of an African evolutionary origin for these tick-borne flaviviruses and their dispersal across the Northern Hemisphere raises concerns over possible spread of a new potentially dangerous infection outside its country of origin. This awareness should alert physicians in Western countries to pathogens that cause unspecific, unusual, or totally unknown clinical signs. We agree that more research is needed in human and animal health, as well as in entomologic and environmental studies, especially in light of the recent data suggesting a nonexclusive role of ticks as vectors for human infection with Alkhurma virus and the hypothesis of human-to-human transmission (*2*).

Past experience with emerging diseases in travelers (Crimean-Congo hemorrhagic fever, Lassa fever, Marburg hemorrhagic fever) or with autochthonous spread of imported diseases (chikungunya, West Nile virus disease, malaria) indicates a consistent delay in the diagnosis of first or sporadic cases, leading to inappropriate or untimely treatment of some of the patients. To confront the problem of unusual and emerging pathogens, Western countries must invest in evidence-based and integrated strategies of preparedness and response.

First, the frontline physicians’ ability to recognize, diagnose, and treat illnesses caused by unusual pathogens should be improved through training covering rare and tropical diseases. Second, a system of timely information and alerts about threats posed by new infectious diseases should be set up. Third, concentrating clinical samples in virology laboratories with proven experience in detecting emerging pathogens is crucial for comprehensive and rapid differential diagnosis. It must be also remembered that no commercial tests are available for serologic or molecular detection of many rare pathogens or for differential diagnosis. And, finally, laboratory diagnosis is often made difficult by antibody cross-reactivity, as documented in our article (*3*).

For Alkhurma virus, further research is needed in the animal setting because little is known about length and severity of illness, duration of viremia, and modes of animal-to-animal and animal-to-human transmission; we need to better understand the role of vectors to limit the spread of the disease.

## References

[1] Charrel RN, Gould EA. Alkhurma hemorrhagic fever in travelers returning from Egypt, 2010 [letter]. Emerg Infect Dis. 2011;17:1573–4.2180166010.3201/eid1708.101858PMC3381587

[2] Madani TA, Azhar EI, Abuelzein el-TM, Kao M, Al-Bar HM, Abu-Araki H, . Alkhumra (Alkhurma) virus outbreak in Najran, Saudi Arabia: epidemiological, clinical, and laboratory characteristics. J Infect. 2011;62:67–76. Epub 2010 Oct 15. 10.1016/j.jinf.2010.09.03220920527

[3] Carletti F, Castilletti C, Di Caro A, Capobianchi MR, Nisii C, Suter F, Alkhurma hemorrhagic fever in travelers returning from Egypt, 2010. Emerg Infect Dis. 2010;16:1979–82.2112223710.3201/eid1612101092PMC3294557
